# Development of a Search Task Using Immersive Virtual Reality: Proof-of-Concept Study

**DOI:** 10.2196/29182

**Published:** 2021-07-02

**Authors:** Samuel Elia Johannes Knobel, Brigitte Charlotte Kaufmann, Stephan Moreno Gerber, Prabitha Urwyler, Dario Cazzoli, René M Müri, Tobias Nef, Thomas Nyffeler

**Affiliations:** 1 Gerontechnology & Rehabilitation Group University of Bern Bern Switzerland; 2 Perception and Eye Movement Laboratory Departments of Neurology and BioMedical Research, Inselspital Bern University Hospital Bern Switzerland; 3 Neurocenter Luzerner Kantonsspital Lucerne Switzerland; 4 Department of Neurology Inselspital, Bern University Hospital University of Bern Bern Switzerland; 5 ARTORG Center for Biomedical Engineering Research University of Bern Bern Switzerland

**Keywords:** virtual reality, serious game, search task, stroke, neglect, usability, development, immersion, concept, gaming

## Abstract

**Background:**

Serious games are gaining increasing importance in neurorehabilitation since they increase motivation and adherence to therapy, thereby potentially improving its outcome. The benefits of serious games, such as the possibility to implement adaptive feedback and the calculation of comparable performance measures, can be even further improved by using immersive virtual reality (iVR), allowing a more intuitive interaction with training devices and higher ecological validity.

**Objective:**

This study aimed to develop a visual search task embedded in a serious game setting for iVR, including self-adapting difficulty scaling, thus being able to adjust to the needs and ability levels of different groups of individuals.

**Methods:**

In a two-step process, a serious game in iVR (bird search task) was developed and tested in healthy young (n=21) and elderly (n=23) participants and in a group of patients with impaired visual exploration behavior (ie, patients with hemispatial neglect after right-hemispheric stroke; n=11). Usability, side effects, game experience, immersion, and presence of the iVR serious game were assessed by validated questionnaires. Moreover, in the group of stroke patients, the performance in the iVR serious game was also considered with respect to hemispatial neglect severity, as assessed by established objective hemispatial neglect measures.

**Results:**

In all 3 groups, reported usability of the iVR serious game was above 4.5 (on a Likert scale with scores ranging from 1 to 5) and reported side effects were infrequent and of low intensity (below 1.5 on a Likert scale with scores ranging from 1 to 4). All 3 groups equally judged the iVR serious game as highly motivating and entertaining. Performance in the game (in terms of mean search time) showed a lateralized increase in search time in patients with hemispatial neglect that varied strongly as a function of objective hemispatial neglect severity.

**Conclusions:**

The developed iVR serious game, “bird search task,” was a motivating, entertaining, and immersive task, which can, due to its adaptive difficulty scaling, adjust and be played by different populations with different levels of skills, including individuals with cognitive impairments. As a complementary finding, it seems that performance in the game is able to capture typical patterns of impaired visual exploration behavior in hemispatial neglect, as there is a high correlation between performance and neglect severity as assessed with a cancellation task.

## Introduction

A crucial component of neurorehabilitation and its success is therapy adherence and repetition. One promising possibility to improve patients' therapy adherence, and thereby its outcome, is to enhance training motivation by means of serious games [1] and immersive virtual reality (iVR) [2,3]. The combination of serious games and iVR allows for the investigation of visual exploration behavior, which is highly relevant in activities of daily living and therefore frequently used in the diagnosis and rehabilitation of patients suffering from a neurodegenerative disease [4-7].

The primary purpose of serious games is not to be fun, but to teach, train, or assess skills in an entertaining way [8]. Due to technological achievements in recent years, their importance in education [9] rehabilitation [10], and medical training [11] is growing, as they have several advantages. First, serious games are standardized, which means that each user will experience the same task. Second, different game settings offer the possibility to automatically adapt the task difficulty to the user’s individual skills and performance level. Finally, serious games offer the possibility to track the user’s game performance (eg, achieved scores) and game behavior as a measure for activities of daily living (eg, reaction times) [12].

The different settings and measured performance values have been used to implement adaptive difficulty scaling, which has shown to be an important element of serious games leading to 2 key elements [13-16]. First, a balance point (ie, when game difficulty is still challenging, yet does not exceed the player's abilities) can be achieved. Second, changes in game mechanics should allow adapting the difficulty throughout the game, to continuously and optimally match the increasing skill level of the player [17]. Importantly, both elements are essential to keep the player motivated [18].

The benefits of serious games can be further improved by the use of iVR [19-21]. iVR presents computer-generated artificial, but interactable (ie, hand-held controllers), 360° environments or pre-rendered 360° videos inside a head-mounted display (HMD). With technological improvements over the last decade, iVR is now also increasingly used in clinical applications such as in motor rehabilitation for gait and balance [22,23], surgery training [24-26], or anxiety treatment [27]. Particularly in tasks that involve any kind of motor activities, iVR has some advantages, such as the possibility to objectively measure progressive improvement in trained skills, perform task-oriented repetitive training, and apply multisensory feedback and task variation [28,29]. Rizzo et al [12] summarized evidence showing that skills gained in the iVR environment can be transferred to activities of daily living (eg, crossing the street [30]) reflecting the ecological validity of tasks in iVR. The high ecological validity can be explained due to fewer distractions from external stimuli and the intuitive interaction with the virtual environment, whereas the interaction and thus behavior in the virtual environment can be tracked by recording head and hand movements. Conclusively, tasks in iVR tend to feel more naturalistic, and several studies have shown that the naturalistic feeling of a task correlates with higher enjoyment, better performance, and better motivation [3,31,32]. This naturalistic feeling is created by the so-called immersion [33-35] (ie, a situation in which the real world is ignored in favor of the virtual environment [36]).

Visual exploration behavior is a crucial element of activities of daily living (eg, crossing the street, grocery shopping) [37] and corresponds to purposefully looking around in the present environment (ie, actively acquiring visual information through coordinated movements of the eyes and head [38]). Therefore, an impaired visual exploration behavior could lead to a reduction of performance in activities of daily living and thus in quality of life [39,40]. Impairment of the visual search behavior is also one landmark of patients suffering from hemispatial neglect [37,41]. Hemispatial neglect is a visuospatial attention disorder that frequently occurs after a right hemispheric stroke. Its characteristic is the inability to attend or respond to stimuli presented within the left contralesional space [42].

Therefore, the aim of this proof-of-concept study was to develop a serious game using iVR in which participants perform a visual search task that encourages the exploration of their environment. We hypothesized that the given task has high usability and limited or no side effects and that the performance can be adapted dynamically to the skills of the participants.

## Methods

The main goal of this study was to develop a gamified search task that encourages players to explore their visual environment. We named it the bird search task, and development and evaluation were divided into 2 steps.

First, the 2D game “Crazy Chicken” (ak tronic Software & Services gmbh, Saerbeck, Germany) was used as inspiration, and the game mechanics were transferred to a 3D iVR environment and then modified and tested with healthy young and elderly participants. Based on the findings of the first step, in the second step, the task was further adapted and tested with patients with hemispatial neglect after a right hemispheric stroke.

### Game Development and Apparatus

#### Bird Search Task for Healthy Participants

The 2D game “Crazy Chicken” was identified as a suitable gamified task because it encourages players to explore their visual environment. In the original “Crazy Chicken” game, the chickens (visual targets) appear at random locations on the computer screen and fly at a constant velocity in random directions. The player, by constantly scrolling left and right on a scrollable, 2D screen, has to search and tag the targets before they disappear after a constant time delay.

**Figure 1:**
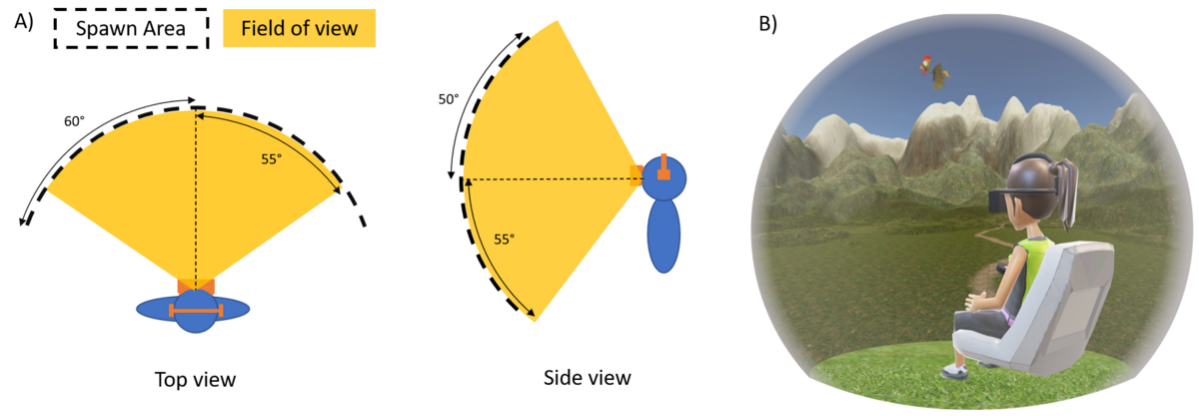
Immersive virtual reality game, with (A) the participant’s field of view (yellow) within the head-mounted display, which moved if the player (blue) turned his or her head, and the area where the target could appear (dashed line; spawn area), which was locked to the midsagittal plane; and (B) a schematic representation of a participant wearing the head-mounted display and performing the task.

**Figure 2:**
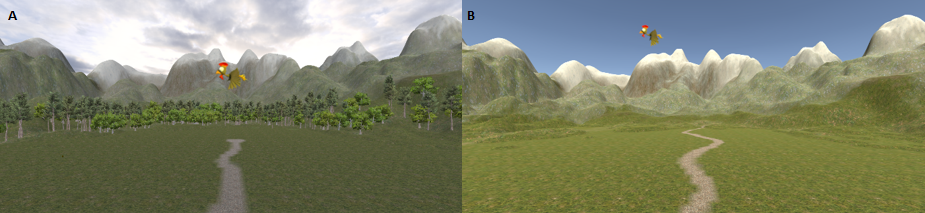
Exemplary scene of the gameplay, (A) as implemented in step 1 (healthy individuals) and (B) after the modifications performed in step 2 (patients with right hemispheric stroke and hemispatial neglect).

To transfer the task into 3D, a simple virtual environment was designed using the gaming development platform Unity3D [43]. The environment was built as a wide circular area surrounded by trees and mountains, as shown in Figure 1 and Figure 2. A mobile gaming laptop was used to render the virtual environment (HP-Omen, graphic-card NVIDIA GTX1050 and CPU Intel i7).

The iVR hardware consisted of an HMD and a hand-held controller (HTC Vive, High Tech Computer Corporation, Taoyuan, Taiwan; resolution of 2160 x 1200 pixels; full HD; horizontal and vertical field of view of 110 degrees [yellow area in Figure 1A]; frame rate of 90 Hz). The x-y position of the handheld controller was continuously recorded. Using iVR hardware, 360° of the virtual environment could be explored by moving the eyes and turning the head. The targets (eg, birds) could be tagged by aligning the handheld controller with the target and simultaneously pressing a trigger on the controller. Target appearance was set to randomly take place within a restricted area in front of the player (± 60° horizontally and ± 50° vertically, as defined with respect to the trunk’s midsagittal plane; see dashed lines in Figure 1A). In order to promote exploration of the whole extent of the virtual environment, the horizontal area in which the targets could appear was 10° larger than the player's field of view (ie, additional 5° on either side; Figure 1A).

To alert the player of a new appearing target, a short (1 second) auditory signal (chicken cackle) was presented binaurally via headphones (XQISIT oE400, Strax Americas Inc, Miami, FL). Then, the target appeared at a position randomly determined within the spawn area and flew horizontally towards the right or left (direction randomly selected) with a constant velocity of 2 °/second. If the target was detected and successfully tagged, it fell vertically and disappeared. In case the target was not tagged within the maximum presentation time of 15 seconds, it disappeared. In either way, after a fixed interstimulus interval of 2 seconds, a new target appeared, again with an alerting cackle.

The game was played in rounds. Each of the 10 played rounds consisted of 30 targets. For each target, the time until it was found was measured, and the percentage of found targets was calculated for each round.

We modified the task further by integrating several difficulty levels, to be able to adapt the task to participants with different needs and skills.

We implemented 15 difficulty levels, in which changes in difficulty were achieved by manipulating both target behavior and task mechanics. For each of the 15 levels, the values of the settings changed stepwise, according to the “Change Per Level” threshold presented in Table 1. Concerning target behavior, both the maximum lifetime and speed of the targets were manipulated across levels. For instance, in the easiest level (Level 1), the targets moved with a speed of 2 °/second and were presented for 15 seconds before they disappeared; in the most difficult level (Level 15), the targets moved with a speed of 35 °/second and were presented for 4 seconds before they disappeared. Hence, the more difficult the level, the faster the participants had to explore the visual environment in order to find the targets before they disappeared. Concerning task mechanics, the task adapted its difficulty (ie, changed the difficulty level) automatically, based on performance in the previous round. Each level had a level-up threshold (ie, if the threshold value was reached, the next round started in a higher difficulty level) and a level-down threshold (ie, if the threshold value was not reached, the next round started in a lower difficulty level), defined according to the percentage of found targets. If the percentage of found targets in a particular round was not higher than the level-up threshold and not lower than the level-down threshold, the difficulty in the next round did not change.

For example, in a round at level 6, the targets move with a speed of 13.8 °/seconds, and the maximum lifetime of the targets is 11.07 seconds. Assuming that the participant would find 25 of the 30 targets in this particular round (ie, 83.3% found targets), then the next round would start at level 7 since the percentage of found targets is above the level-up threshold of level 6 (ie, 77.1%). If the player finds only 15 of the 30 targets (ie, 50% found targets), then the next round would start at level 5, since the percentage of found targets would be below the level-down threshold of level 6 (ie, 67.1%).

**Table 1:** Change per level algorithm.

Task and target parameters	Level 1	Change per level^a^	Level 15
Lifetime (seconds)	15	–0.786	4
Speed (°/second)	2	+2.36	35
Level-up threshold (%)	>70	+1.42	>90
Level-down threshold (%)	<60	+1.42	<80

^a^The stepwise change per level between Level 1 and Level 15.

#### Bird Search Task for Patients With Hemispatial Neglect

In a second step, based on the findings collected in healthy participants and on recommendations of the clinicians, the version for patients was developed. As neglect's clinical picture is very heterogeneous across patients, the task needs to be easily adaptable to the patients' individual needs. The aim was that patients would not get overwhelmed and frustrated due to too great a difficulty but also not get bored by a task that was too easy. For this purpose, we implemented an easy-to-use setting file, in which the total number of targets, number of targets per round, and starting difficulty level could be set individually for each patient. Additionally, several adaptations in design and task mechanics were performed. First, as patients with neglect process visual information slower [44,45], the landscape of the gameplay would be too complex; therefore, in order to reduce the number of distractive elements, the trees in the background and the clouds in the sky were removed from the game scenery (see Figure 2). Second, based on the recommendations of clinicians, the minimal speed of the targets was lowered from 2 °/second to 0.1 °/second; this manipulation generally lowered the difficulty level as well as reduced the need for fast head movements that could promote side effects.

### Participants

The study was approved by the Ethics Committee of the Cantons of Bern and the Ethics Committee of north-west and central Switzerland and was conducted in accordance with the latest version of the Declaration of Helsinki. All participants gave written informed consent before participation.

In step one, the feasibility of the task was assessed with 21 younger healthy participants recruited at the University of Bern (10 women; mean age, 28.1, SD 5.5 years) and with 23 older healthy participants recruited during a chess tournament for seniors (1 woman; mean age, 71.3, SD 6.3 years). All participants had no history of neurological nor psychiatric disorders. Previous VR experience was reported by 16 of the younger participants and 1 of the older participants.

In step two, 11 inpatients with hemispatial neglect (5 women; mean age, 69.6, SD 13.0 years) after right hemispheric, subacute stroke were recruited at the Neurorehabilitation Clinics of the Inselspital, Bern University Hospital (sites Bern and Riggisberg) and of the Kantonsspital Luzern, Switzerland. Demographic characteristics for each patient are presented in Table 2. The study was always conducted with a mobile setup in the place where the patient was currently hospitalized. All patients showed significant left-sided neglect in activities of daily living, as assessed with the Catherine Bergego Scale (CBS, Range 0-30, 0 = normal) [46], and had normal or corrected-to-normal vision. One of the patients reported previous VR experience.

**Table 2:** Individual demographical and neuropsychological data from step 2 for patients with hemispatial neglect.

Patient code	Age range (years)	Gender	Lesion type	Time since stroke (days)	CBS^a^	CoC^b^ (SNT^c^ single)	Number of played chickens	Play duration (min)
P_26	70-75	Male	Ischemic	85	6	0.184	195	16.5
P_27	55-60	Male	Hemorrhagic	145	2	0.208	100	10.4
P_28	80-85	Male	Bleeding	49	8	0.746	50	9.4
P_29	80-85	Female	Ischemic	52	9	0.998	44	8.1
P_30	75-80	Male	Ischemic	42	8	0.293	80	9.2
P_31	50-55	Female	Ischemic	106	3	0.414	80	13.2
P_32	65-70	Female	Ischemic	59	2	–0.067	80	10.2
P_33	70-75	Female	Ischemic	58	3	0.824	80	9.3
P_34	85-90	Male	Ischemic	29	18	0.998	80	8.4
P_35	50-55	Male	Hemorrhagic	39	10	0.046	150	13.0
P_36	60-65	Female	Ischemic	38	17	0.191	100	10.1

^a^CBS: Catherine Bergego Scale (0-30).

^b^CoC: Center of Cancellation (–1 to 1).

^c^SNT: Sensitive Neglect Test.

In addition to CBS, where a value ≥1 means the patient has a neglect, the objective neglect severity was assessed on the day of the task by means of the paper-and-pencil Sensitive Neglect Task (SNT), single version [47]. The SNT is a cancellation task in which patients are asked to mark 40 targets among 240 distractors. Based on the distribution of the marked targets, the Center of Cancellation (CoC) [48] was computed, representing an objective measure of neglect severity. The CoC reflects the mean deviation of the marked targets from the center and is normalized to values ranging from –1 to 1. Zero indicates no spatial bias, where a CoC ≥0.081 represents a significant rightward shift (ie, left-sided neglect).

### Outcome Measures

#### Questionnaires

To evaluate the feasibility and usability of the newly implemented task, several questionnaires were used to assess the participants' individual gaming experience.

To assess acceptance, usability, and participant's perception of the visual search task and of the VR system, 3 questions from the System Usability Scale (SUS) [49] were used, as previously reported by Gerber et al [50] and Knobel et al [51]. The questions were answered using a 5-point Likert-scale, ranging from “fully disagree” to “fully agree.” The mean score across all questions was calculated for each participant.

The Simulator Sickness Questionnaire (SSQ) [52] was used to assess side effects such as cybersickness, oculomotor problems, and disorientation [53,54]. In order to reduce the workload of the study participants, a subset of 7 questions from the SSQ was used, as previously reported by Gerber et al [50] and Knobel et al [51]. The questions were answered using a 4-point Likert-scale (ie, “None,” “Slight,” “Moderate,” “Severe”). Again, the mean score across all questions was calculated for each participant.

Additionally, in order to assess the enjoyment of the task, the Perception of Game Training Questionnaire (PGTQ) [55] was administered. The PGTQ consists of 4 questions that are answered using a 7-point Likert-scale, ranging from “fully disagree” to “fully agree.” Each question represented a different aspect of the perception of the task (ie, motivation, frustration, how challenging it is, entertainment). Therefore, no mean score was calculated across questions; instead, each score was considered independently.

Furthermore, to assess immersion and presence, questions from the Igroup Presence Questionnaire (IPQ) [56] were used. The question subset was already used in another iVR study by Gerber et al [50] and was answered on a 5-point Likert-scale.

The questions from each questionnaire are shown in Table 3.

**Table 3:** Exact formulations that were asked in the questionnaires.

Questionnaire, number		Question		Domain	
**SUS^a,b^**					
	1	I thought the system was easy to use		Usability	
	2	I think that I would like to use this system frequently		Usability	
	3	I felt very confident using the system		Usability	
**SSQ^c,d^**					
	4	General discomfort		Sickness	
	5	Stomach awareness		Sickness	
	6	Sweating		Sickness	
	7	Nausea		Sickness	
	8	Headache		Oculomotor problems	
	9	Eye strain		Oculomotor problems	
	10	Dizziness		Disorientation	
**IPQ^e,f^**					
	11	In the virtual world, I had a sense of “being there.”^g^		Immersion	
	12	Somehow, I felt that the virtual world surrounded me.^h^		Presence	
**PGTQ^i,j^**					
	13	I was motivated for a good performance.		Motivation	
	14	The game was frustrating.		Frustration	
	15	The game was challenging.		Challenge	
	16	The game was entertaining.		Entertainment	

^a^SUS: System Usability Scale.

^b^Fully disagree to fully agree; score range, 1-5; midpoint, 3; scored as the mean of Q1-Q3.

^c^SSQ: Simulator Sickness Questionnaire.

^d^None to severe; score range, 1-4; midpoint, 2.5; scored as the mean of Q4-Q10.

^e^IPQ: Igroup Presence Questionnaire.

^f^Score range, 1-5; midpoint, 3; each question is scored individually.

^g^Not at all to very much.

^h^Fully disagree to fully agree.

^i^PGTQ: Perception of Game Training Questionnaire.

^j^Fully disagree to fully agree; score range, 1-7; midpoint, 4.5; each question is scored individually.

#### Performance Indicators During the Task

The presented task allowed us to measure several performance indicators, namely the changes in difficulty levels over time and the mean search time to detect the targets.

Based on the mean search time of the targets, the mean search time per participant was calculated, representing the mean time until a participant tagged a target (not-found targets were excluded). Moreover, the controller position of the VR setup was recorded over the entire task, which allowed us to track the participants’ hand positions over time, hence delivering information concerning their spatial search behavior.

#### Evaluation of the Difficulty Scaling

There are different possibilities to assess whether an adaptive difficulty scaling is successful. An easy indirect, but less objective, possibility is to simply evaluate the results of the questionnaires concerning entertainment and frustration. A more elaborate and objective approach is to consider the number of level changes over the task rounds with respect to the starting level. A population in which the starting level is much easier than the average balance point should show a greater increase in difficulty (ie, more upward level switches) in the initial phases of the task (ie, when they increase to their balance point) and then a smaller increase over time (ie, when the balance point is reached, but the participants still gradually get better at the task due to practice). The better the starting level matches the abilities of the group, the smaller the difficulty changes should be in the initial phases of the task. Nevertheless, due to practice (eg, better aiming, better search strategies), participants are expected to get better in the task, and there should thus be at least a small difficulty level increase over time.

A plateau reflects the balance point at which the level did not change anymore between rounds. This indicates that participants reached their optimal task difficulty level (ie, their performance was not at the ceiling in a particular difficulty level so that the algorithm would increase it in the next round and not at the floor that the algorithm would decrease the difficulty level in the next round).

### Statistical Analyses

The mean SUS and SSQ scores, reflecting the usability and number of side effects, respectively, were computed for each group (ie, young, elderly, and stroke). The 3 and 7 items of the SUS and SSQ, respectively, were averaged.

The means of the groups for the PGTQ and the IPQ questions were calculated per question and were displayed as histograms.

The change in level for every participant was computed by subtracting the starting level from the levels they were in the consecutive rounds. This change of level was used as a performance measure and visualized as the level change over time relative to the starting level. The position over time of the hand was illustrated by plotting the controller x-y position over time.

The difference in search time between the young group and elderly group was calculated using a 2-sided *t* test. For the neglect group, a Pearson correlation was calculated between neglect severity (CoC in stroke group) and the mean search time of targets in the VR game. For the statistical analysis, the alpha was set to 0.05.

All analyses and visualizations were performed with R [57] and MATLAB [58].

## Results

### Questionnaires

In step one, the feasibility and usability of the task were assessed in 2 groups: young and elderly healthy participants. The analysis of the SUS scores (mean of the 3 questions; see Table 3) revealed high usability in both groups (Table 4). Both young and elderly participants reported that they would even like to play the task frequently (mean scores: young, 3.95, SD 0.74; elderly, 4.04, SD 1.26).

In general, almost no side effects were reported, as assessed with the SSQ score (mean of the 7 domains; see Table 3). In the young group, the mean score reflected minimal side effects (Table 4). More precisely, only 3 young participants reported severe side effects in 1 of the 7 domains (2 cases of stomach awareness and 1 case of dizziness). In the elderly group, the mean score was similarly low (Table 4). Only 1 elderly participant reported a severe side effect (sweating).

The PGTQ consisted of 4 questions (for details, see Table 3) concerning motivation, frustration, challenge, and entertainment. The participants of both groups were very motivated (mean scores: young, 6.38, SD 1.12; elderly, 6.73, SD 0.46), entertained (mean scores: young, 6.28, SD 0.56; elderly, 5.83, SD 1.70), and not frustrated (mean scores: young, 2.57, SD 0.90; elderly, 1.83, SD 1.44). The extent to which the task was challenging was rated above midline in both groups (mean scores: young, 4.29, SD 0.90; elderly, 5.61, SD 1.23).

**Figure 3:**
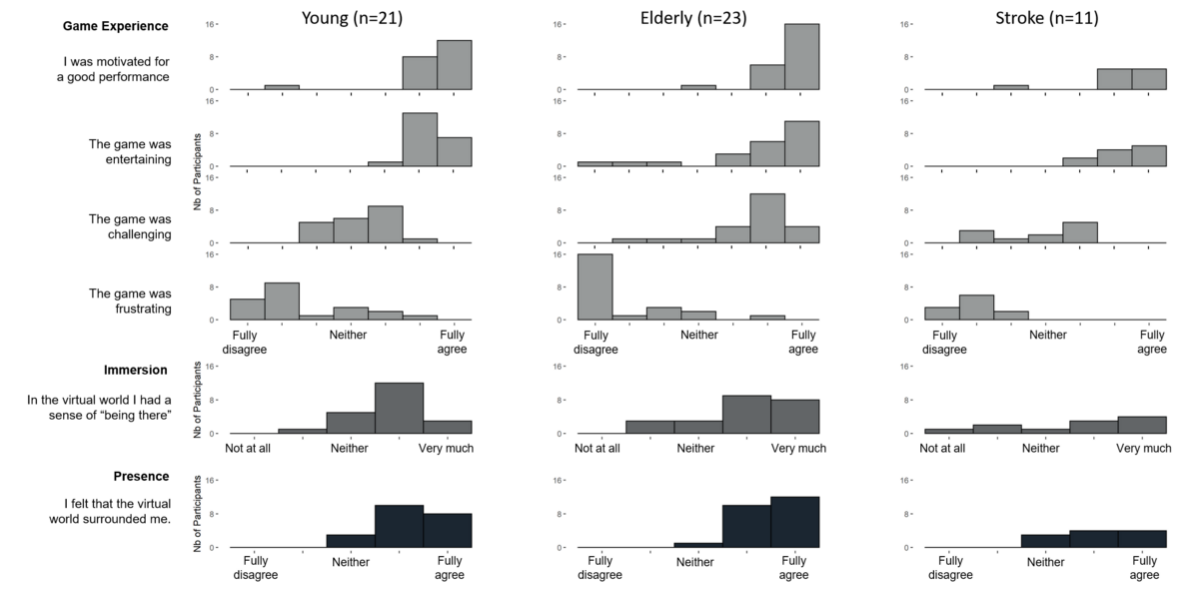
Igroup Presence Questionnaire results from the healthy participants (young, elderly) and the patients (stroke).

The question for immersion (mean scores: young, 3.81, SD 0.75; elderly, 3.96, SD 1.02) and presence (mean scores: young, 4.24, SD 0.70; elderly, 4.48, SD 0.59) were rated high in both healthy groups (Figure 3).

In step two, the feasibility of the task was assessed in patients with neglect. Patients with neglect rated the task as highly usable (Table 4), and almost no side effects were reported on the SSQ (Table 4). More precisely, none of the patients reported any severe side effect. Only 3 patients reported side effects. One patient reported moderate sweating, while 2 others reported mild sweating and mild headache.

According to the PGTQ scores (Figure 3), the patients were highly motivated (mean 6.18, SD 1.17), entertained (mean 6.27, SD 0.79), and not frustrated (mean 1.91, SD 0.70). Furthermore, the degree of challenging score was around the midline (mean 3.82, SD 1.33; ie, most patients rated the task as neither too difficult nor too easy). The patients felt high immersion (mean 3.64, SD 1.43) and presence (mean 4.09, SD 0.83)

**Table 4:** Results of the System Usability Scale (SUS), where 1 means “unusable” and 5 means “very usable,” and the Simulator Sickness Questionnaire (SSQ), where 1 means “None” and 4 means “Severe” side effects.

Study group	SUS, mean (SD)	SSQ, mean (SD)
Young	4.41 (0.49)	1.42 (0.45)
Elderly	4.46 (0.73)	1.27 (0.24)
Stroke	4.73 (0.44)	1.05 (0.10)

### Adaptive Difficulty Scaling

**Figure 4:**
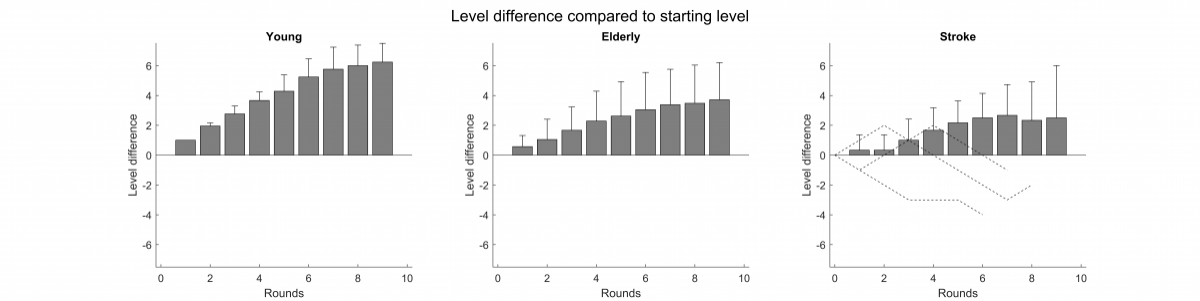
Level difference (and standard error) in the number of levels over the round compared to the starting level. In the neglect-group for the bars, only the 6 patients that played for 10 rounds were included. The raw values are included for the other 3 patients. The level difference is the mean of the difference between the level in round x minus the starting level.

In all 3 groups, the qualitative illustration of the increase in the level differences shows a plateau over time (Figure 4).

In young participants, a steep increase in the level difference (ie, the levels get more difficult) was found at the beginning of the task, reaching a plateau (optimum difficulty) at a level that was close to the possible maximum of 7 increases. There were only 7 increases possible because they started at level 8, meaning they could level up to 15 by increasing their level 7 times.

In elderly participants and stroke patients, the increase in the level difference revealed a more moderate and heterogeneous change over time. Both groups needed more rounds to reach their optimum; compared visually, this optimum was lower than in the young group.

### In-Game Measures

#### Controller Position Over Time

**Figure 5:**
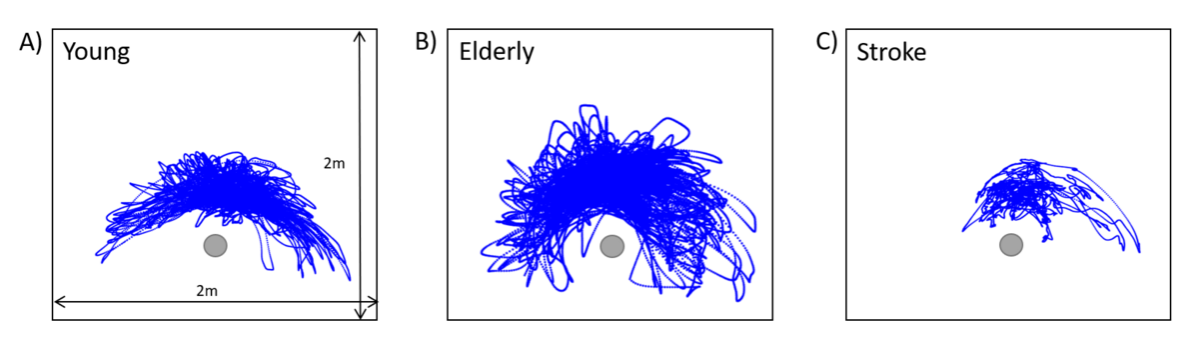
The 3 panels show 3 exemplary controller movements (within a 2x2 m space), which represents the participant's hand movement around the participant’s position (grey circle) over the total course of the task.

During the task, the controller position was continuously recorded and could be extracted for offline analysis. Figure 5 presents the exemplary data of the controller position (highlighted in blue) for one participant per group.

In the exemplary participants of the young group (Figure 5A) and the elderly group (Figure 5B), the controller movements were symmetrically distributed. In the exemplary participants of the stroke group (Figure 5 C), a narrowing of the movement as well as a clear rightward shift can be observed: The patients’ hand movements mainly took place within the right hemispace due to left neglect.

#### Search Time

**Figure 6:**
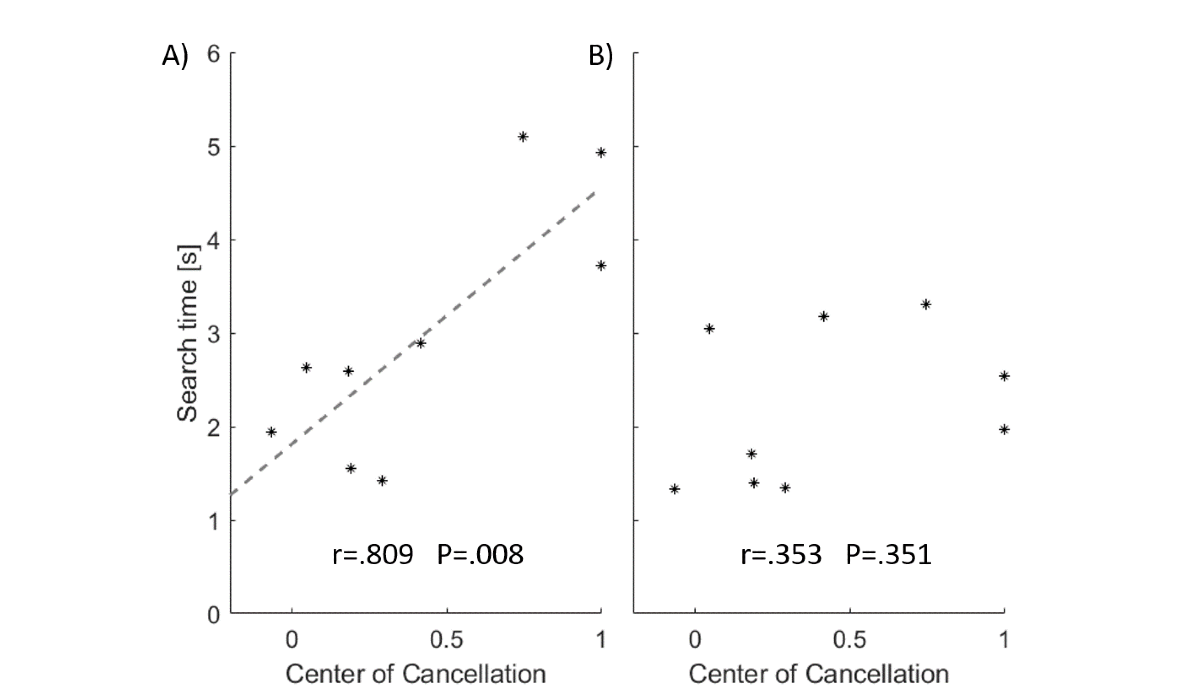
Correlation between the Center of Cancellation (CoC; as an objective measure of neglect severity) in the Sensitive Neglect Test (SNT) and the mean search time for the (A) left hemispace and (B) right hemispace in patients with neglect.

The mean search time in young (mean 1.533, SD 0.342) and elderly (mean 1.694, SD 0.275) participants did not significantly differ (*t*_38.4_=1.71, *P*=.095). There was a significant, strong correlation between the mean search time and neglect severity (ie, the CoC in the SNT; *r*=0.70, *P*=.037; [59]). An additional analysis investigating the correlation between search time per hemispace (ie, targets appearing within the left vs right hemispace) and neglect severity revealed a hemispace-dependent effect (Figure 6). A significant correlation was found for targets appearing within the left hemispace (*r*=.809, *P*=.008; strong correlation [59]). However, for targets appearing within the right hemispace, no significant correlation was found (*r*=.353, *P*=.351).

## Discussion

For this study, we developed and evaluated a dynamic visual search task in iVR. As hypothesized, the task could easily be adapted to different skills, impairments, and experience levels. Further, the developed system has high usability and acceptance and resulted in only slight or no side effects in healthy participants and in right-hemispheric stroke patients with hemispatial neglect.

### Questionnaires and Feedback

The ratings for usability and acceptance of the bird search task were high in both healthy participants and stroke patients with hemispatial neglect. This result is in line with previous findings from studies investigating the acceptance of HMD-VR in elderly [60,61], studies using VR for neglect diagnostics [51], and studies in which a similar VR setup was used to apply audio-visual stimulation in critically ill patients [62].

Regarding side effects, overall, only slight to none were reported. Furthermore, the nature and intensity of side effects were in line with the literature [51,54,62]. In the group of patients with neglect, no severe side effects were reported (only 1 participant reported moderate sweating). This is potentially due to the game adaptations (eg, slower targets require slower head movements), simplification, and individual adjustments performed for the patients in step two. This low rate of side effects is also in line with the high values for immersion and presence, as those correlate negatively [63].

The game experience was assessed by means of a questionnaire with 4 parts (frustration, degree of challenge, entertainment, motivation). Indeed, motivation, entertainment, and low frustration levels are crucial aspects for any possible future application [64]. In the bird search task, all participants were highly motivated, entertained, and even though not all participants found all targets, they did not feel frustrated. In particular, the group of patients with neglect had high levels of motivation and entertainment and low frustration levels due to 2 possible reasons. First, this might be the result of the adaptive difficulty scaling; second, this could be the result of the ability to individualize the starting conditions to the patients' needs and abilities. According to the literature, high motivation is also in line with the high values for immersion the participants reported [31,32,65,66].

Interestingly, the question as to whether the task was challenging was answered very differently among the 3 groups. While elderly healthy participants reported the task to be very challenging, the young healthy participants did find it rather easy. This difference between the elderly and young groups is not surprising, as most of the participants in the elderly group did not have any VR experience, whereas the young group was rather experienced in VR. The distribution of answers in patients with neglect was rather spread, indicating that, for some patients, the task was not particularly challenging, while for others, it was. A possible explanation for this result might be found in the individual settings adapted for the group of patients with neglect. As the individual performance level was estimated, some might have been under- or overestimated. Nevertheless, even if the performance level of some patients with neglect would have been under-or overestimated, according to their ratings, they were still highly entertained and motivated.

### Difficulty Levels and Adaptive Difficulty Scaling

The descriptive analysis of the change in difficulty levels over time evidenced that the participants in the young group showed a steep increase in the initial phases of the task, suggesting a higher balance point than the one they started with. In the elderly group, the initial increase was less steep, but it was still clearly observable and gradual over time. In the neglect group, the initial increase seems to be delayed, but over time, there is a level increase relative to the starting level. This might be due to the attentional impairments and other cognitive deficits, resulting in a need for more rounds to get better at the game. Nevertheless, based on this measure, we could show that the individualized task was able to adapt to the individual level of impairment of the patients over time and thus keep them motivated and not overwhelmed [18].

Therefore, as the feedback regarding motivation and frustration assessed by means of the questionnaires shows clearly, the adaptive difficulty scaling was able to address the issue of very different skill levels and different progression speeds across participants.

### In-Game Measures

VR tasks offer several opportunities to individually evaluate participants' task performance using in-game measures. One of these possibilities is the analysis of the hand position over time, reflecting the spatial search behavior of the participants. As patients with neglect typically fail to explore the contralesional space, the assessment of the individual hand movements may be a valuable parameter to characterize neglect manifestations and severity. Indeed, in exemplary data sets, we were able to show typical neglect patterns (ie, healthy participants move their hand in the peripersonal space symmetrically during the search, whereas for patients with neglect, these movements are limited within the left, contralesional side).

Another parameter to estimate the ecological validity of the task in patients with neglect is the average search time relative to the objective neglect severity. Our results revealed the typical neglect pattern [37,67]; the worse the neglect — as measured by the CoC — the more time the stroke patients needed to find targets appearing within their contralesional, left side.

### Limitations

The main limitation of the present study is the small sample size of the neglect group and the fact that the patients were not assessed with a comprehensive test battery (ie, including other measures of neglect severity like behavior in free visual exploration [6], other cancellation tasks [68], or line bisection tasks [69]). Furthermore, even though the group was age-matched, there were mainly male participants in the group of healthy elderly participants, and as they were all chess players, the generalizability of the results in this group are limited. For this, further research with a more representative sample would be needed.

Due to the 2-step process, no direct comparison of the in-game performance between participants with normal and impaired visual exploration behavior was possible.

### Outlook and Conclusion

The presented bird search task was shown to be entertaining, motivating, and even immersive. Due to the implemented difficulty levels, it adapted well to different populations with different skills and previous VR experience and even in patients with cognitive disturbance after stroke. In particular, the bird search task seems to be able to pick up on typical patterns of neglect and to correlate with the results of established instruments.

Future studies should investigate and evaluate these aspects for potential application in diagnosis or therapy. For the further evaluation of the potential diagnostic or rehabilitative value, the frequency and duration of playing the task should be investigated in a longitudinal randomized clinical trial. Furthermore, the tool should be compared with the standard care of patients with neglect.

## Abbreviations

CBS: Catherine Bergego Scale
CoC: Center of Cancellation
HMD: head-mounted display
IPQ: Igroup Presence Questionnaire
iVR: immersive virtual reality
PGTQ: Perception of Game Training Questionnaire
SNT: Sensitive Neglect Test
SSQ: Simulator Sickness Questionnaire
SUS: System Usability Scale

## References

[1] Sokolov AA, Collignon A, Bieler-Aeschlimann M (2020). Serious video games and virtual reality for prevention and neurorehabilitation of cognitive decline because of aging and neurodegeneration. Curr Opin Neurol.

[2] Rizzo A, Kim GJ (2005). A SWOT Analysis of the Field of Virtual Reality Rehabilitation and Therapy. Teleoperators and Virtual Environments.

[3] Witmer BG, Singer MJ (1998). Measuring Presence in Virtual Environments: A Presence Questionnaire. Presence.

[4] Paladini RE, Wyss P, Kaufmann B, Urwyler P, Nef T, Cazzoli D, Nyffeler T, Müri RM (2019). Re-fixation and perseveration patterns in neglect patients during free visual exploration. Eur J Neurosci.

[5] Pflugshaupt T, Bopp S, Heinemann D, Mosimann U, von Wartburg R, Nyffeler T, Hess CW, Müri RM (2004). Residual oculomotor and exploratory deficits in patients with recovered hemineglect. Neuropsychologia.

[6] Kaufmann BC, Knobel S, Nef T, Müri RM, Cazzoli D, Nyffeler T (2019). Visual Exploration Area in Neglect: A New Analysis Method for Video-Oculography Data Based on Foveal Vision. Front Neurosci.

[7] Kerkhoff G, Keller I, Artinger F, Hildebrandt H, Marquardt C, Reinhart S, Ziegler W (2012). Recovery from auditory and visual neglect after optokinetic stimulation with pursuit eye movements--transient modulation and enduring treatment effects. Neuropsychologia.

[8] Michael D, Chen S (2005). Serious games: games that educate, train and inform.

[9] Ellaway R (2011). Reflecting on multimedia design principles in medical education. Med Educ.

[10] Sabri H, Cowan B, Kapralos B, Porte M, Backstein D, Dubrowskie A (2010). Serious games for knee replacement surgery procedure education and training. Procedia - Social and Behavioral Sciences.

[11] Laamarti F, Eid M, El Saddik A (2014). An Overview of Serious Games. International Journal of Computer Games Technology.

[12] Rizzo AA, Schultheis M, Kerns K, Mateer C (2004). Analysis of assets for virtual reality applications in neuropsychology. Neuropsychological Rehabilitation.

[13] Barzilay O, Wolf A (2009). A Virtual Adaptive Biofeedback System for Neuromuscular Rehabilitation.

[14] Cameirao MS, Bermudez i Badia S, Oller ED, Verschure PFMJ (2008). Using a Multi-Task Adaptive VR System for Upper Limb Rehabilitation in the Acute Phase of Stroke.

[15] Ma M, McNeill M, Charles D, McDonough S, Crosbie J, Oliver L, McGoldrick C, Stephanidis C (2007). Adaptive Virtual Reality Games for Rehabilitation of Motor Disorders. Universal Access in Human-Computer Interaction. Ambient Interaction. UAHCI 2007. Lecture Notes in Computer Science, vol 4555.

[16] Vaughan N, Gabrys B, Dubey V (2016). An overview of self-adaptive technologies within virtual reality training. Computer Science Review.

[17] Sherry Jl (2004). Flow and Media Enjoyment. Commun Theory.

[18] Zohaib M (2018). Dynamic Difficulty Adjustment (DDA) in Computer Games: A Review. Advances in Human-Computer Interaction.

[19] Rizzo AS, Koenig ST (2017). Is clinical virtual reality ready for primetime?. Neuropsychology.

[20] Tieri G, Morone G, Paolucci S, Iosa M (2018). Virtual reality in cognitive and motor rehabilitation: facts, fiction and fallacies. Expert Rev Med Devices.

[21] Chang TP, Sherman J, Gerard J, Nestel D, Hui J, Kunkler K, Scerbo MW, Calhoun AW (2019). Overview of Serious Gaming and Virtual Reality. Healthcare Simulation Research.

[22] Cano Porras D, Siemonsma P, Inzelberg R, Zeilig G, Plotnik M (2018). Advantages of virtual reality in the rehabilitation of balance and gait: Systematic review. Neurology.

[23] Keshner EA, Weiss PT, Geifman D, Raban D (2019). Tracking the evolution of virtual reality applications to rehabilitation as a field of study. J Neuroeng Rehabil.

[24] Torkington J, Smith SG, Rees BI, Darzi A (2001). Skill transfer from virtual reality to a real laparoscopic task. Surg Endosc.

[25] Nemani A, Ahn W, Cooper C, Schwaitzberg S, De S (2018). Convergent validation and transfer of learning studies of a virtual reality-based pattern cutting simulator. Surg Endosc.

[26] Bongers PJ, Diederick van Hove P, Stassen LPS, Dankelman J, Schreuder HWR (2015). A new virtual-reality training module for laparoscopic surgical skills and equipment handling: can multitasking be trained? A randomized controlled trial. J Surg Educ.

[27] Oing T, Prescott J (2018). Implementations of Virtual Reality for Anxiety-Related Disorders: Systematic Review. JMIR Serious Games.

[28] Keshner EA, Fung J (2017). The quest to apply VR technology to rehabilitation: tribulations and treasures. VES.

[29] Levin MF, Weiss P, Keshner E (2015). Emergence of virtual reality as a tool for upper limb rehabilitation: incorporation of motor control and motor learning principles. Phys Ther.

[30] Kim DY, Ku J, Chang WH, Park TH, Lim JY, Han K, Kim IY, Kim SI (2010). Assessment of post-stroke extrapersonal neglect using a three-dimensional immersive virtual street crossing program. Acta Neurol Scand.

[31] Engeser S, Rheinberg F, Vollmeyer R, Bischoff J (2005). Motivation, Flow-Erleben und Lernleistung in universitären Lernsettings 1Dieser Beitrag wurde unter der geschäftsführenden Herausgeberschaft von Joachim C. Brunstein akzeptiert. Zeitschrift für Pädagogische Psychologie.

[32] Lombard M, Ditton T (1997). At the Heart of It All: The Concept of Presence. Journal of Computer-Mediated Communication.

[33] Pratt D, Zyda M, Kelleher K (1995). Virtual Reality: In the Mind of the Beholder. Naval Postgraduate School: Dudley Knox Library.

[34] Székely G, Satava R (1999). Virtual reality in medicine. Interview by Judy Jones. BMJ.

[35] Riva G (2005). Virtual reality in psychotherapy: review. Cyberpsychol Behav.

[36] Costello PJ, Advisory Group on Computer Graphics (1997). Health and safety issues associated with virtual reality: a review of current literature. Technical report series (Advisory Group on Computer Graphics).

[37] Müri RM, Cazzoli D, Nyffeler T, Pflugshaupt T (2009). Visual exploration pattern in hemineglect. Psychol Res.

[38] Franchak JM, Federmeier KD, Schotter ER (2020). Chapter Three - Visual exploratory behavior and its development. Psychology of Learning and Motivation.

[39] Buxbaum LJ, Ferraro MK, Veramonti T, Farne A, Whyte J, Ladavas E, Frassinetti F, Coslett HB (2004). Hemispatial neglect: Subtypes, neuroanatomy, and disability. Neurology.

[40] Jehkonen M, Laihosalo M, Kettunen JE (2006). Impact of neglect on functional outcome after stroke: a review of methodological issues and recent research findings. Restor Neurol Neurosci.

[41] Sprenger A, Kömpf D, Heide W (2002). Visual search in patients with left visual hemineglect. Prog Brain Res.

[42] Heilman KM, Valenstein E (1981). Clinical neuropsychology. Ann Neurol.

[43] Unity Technologies.

[44] Husain M, Rorden C (2003). Non-spatially lateralized mechanisms in hemispatial neglect. Nat Rev Neurosci.

[45] Cazzoli D, Kaufmann BC, Paladini RE, Müri RM, Nef T, Nyffeler T (2021). Anterior insula and inferior frontal gyrus: where ventral and dorsal visual attention systems meet. Brain Commun.

[46] Bergego C, Azouvi P, Samuel C, Marchal F, Louis-Dreyfus A, Jokic C, Morin L, Renard C, Pradat-Diehl P, Deloche G (1995). Validation d'une échelle d'évaluation fonctionnelle de l'héminégligence dans la vie quotidienne: l'échelle CB. Annales de Réadaptation et de Médecine Physique.

[47] Reinhart S, Leonhard E, Kerkhoff G Sensitive Neglect Test (SNT) single and dual task. Saarland University.

[48] Rorden C, Karnath H (2010). A simple measure of neglect severity. Neuropsychologia.

[49] Brooke J (1996). SUS - A quick and dirty usability scale.

[50] Gerber SM, Jeitziner M, Wyss P, Chesham A, Urwyler P, Müri RM, Jakob SM, Nef T (2017). Visuo-acoustic stimulation that helps you to relax: A virtual reality setup for patients in the intensive care unit. Sci Rep.

[51] Knobel SEJ, Kaufmann B, Gerber S, Cazzoli D, Müri RM, Nyffeler T, Nef T (2020). Immersive 3D Virtual Reality Cancellation Task for Visual Neglect Assessment: A Pilot Study. Front Hum Neurosci.

[52] Kennedy RS, Lane NE, Berbaum KS, Lilienthal MG (1993). Simulator Sickness Questionnaire: An Enhanced Method for Quantifying Simulator Sickness. The International Journal of Aviation Psychology.

[53] LaViola JJ (2000). A discussion of cybersickness in virtual environments. SIGCHI Bull.

[54] Huygelier H, Schraepen B, Lafosse C, Vaes N, Schillebeeckx F, Michiels K, Note E, Vanden Abeele V, van Ee R, Gillebert CR (2020). An immersive virtual reality game to train spatial attention orientation after stroke: A feasibility study. Appl Neuropsychol Adult.

[55] Boot WR, Champion M, Blakely DP, Wright T, Souders DJ, Charness N (2013). Video games as a means to reduce age-related cognitive decline: attitudes, compliance, and effectiveness. Front Psychol.

[56] Schubert T, Friedmann F, Regenbrecht H, Paton R, Neilson I (1999). Embodied Presence in Virtual Environments. Visual Representations and Interpretations.

[57] R Core Team (201). R: A Language and Environment for Statistical Computing.

[58] MATLAB. MathWorks.

[59] Cohen J (1988). Statistical Power Analysis for the Behavioral Sciences.

[60] Huygelier H, Schraepen B, van Ee R, Vanden Abeele V, Gillebert C (2019). Acceptance of immersive head-mounted virtual reality in older adults. Sci Rep.

[61] Cook N, Winkler S (2016). Acceptance, Usability and Health Applications of Virtual Worlds by Older Adults: A Feasibility Study. JMIR Res Protoc.

[62] Gerber SM, Jeitziner M, Knobel S, Mosimann U, Müri RM, Jakob S, Nef T (2019). Perception and Performance on a Virtual Reality Cognitive Stimulation for Use in the Intensive Care Unit: A Non-randomized Trial in Critically Ill Patients. Front Med (Lausanne).

[63] Weech S, Kenny S, Barnett-Cowan M (2019). Presence and Cybersickness in Virtual Reality Are Negatively Related: A Review. Front Psychol.

[64] Wüest S, van de Langenberg R, de Bruin ED (2014). Design considerations for a theory-driven exergame-based rehabilitation program to improve walking of persons with stroke. Eur Rev Aging Phys Act.

[65] Kim SY, Prestopnik N, Biocca F (2014). Body in the interactive game: How interface embodiment affects physical activity and health behavior change. Computers in Human Behavior.

[66] Weibel D, Wissmath B (2011). Immersion in Computer Games: The Role of Spatial Presence and Flow. International Journal of Computer Games Technology.

[67] Deouell LY, Sacher Y, Soroker N (2005). Assessment of spatial attention after brain damage with a dynamic reaction time test. J. Inter. Neuropsych. Soc.

[68] Gauthier L, Dehaut F, Joanette Y (1989). The Bells Test: A Quantitative and Qualitative Test For Visual Neglect. International Journal of Clinical Neuropsychology.

[69] Schenkenberg T, Bradford D, Ajax E (1980). Line bisection and unilateral visual neglect in patients with neurologic impairment. Neurology.

